# Left bundle branch block morphology on electrocardiogram after massive diphenhydramine overdose

**DOI:** 10.1016/j.toxrep.2025.102029

**Published:** 2025-04-22

**Authors:** Kara Yeung, Riku Moriguchi, Jeremy Hardin, Henrik Galust, Mina Ghobrial, Martin Krause, Justin Seltzer, Richard Clark

**Affiliations:** a Division of Medical Toxicology, Department of Emergency Medicine, UC San Diego Health, CA, USA; b VA San Diego Healthcare System, San Diego, CA, USA; c San Diego Division, California Poison Control System, San Diego, CA, USA; d Division of Critical Care Medicine, Department of Emergency Medicine, UC San Diego Health, CA, USA; e Division of Anesthesia Critical Care Medicine, Department of Anesthesia, UC San Diego Health, CA, USA; f Southern California Permanente Medical Group, San Diego, CA, USA; g Eisenhower Health, Rancho Mirage, CA, USA

**Keywords:** Diphenhydramine, Cardiotoxicity, Left bundle branch block, Sodium channel blockade, EKG

## Abstract

Diphenhydramine is a first-generation antihistamine with various pharmacologic effects including sodium channel blockade. Electrocardiographic findings often presents with QRS prolongation with a rightward axis. Left bundle branch block (LBBB) is a rare manifestation in this context. We present a case of a 35-year-old woman who presented to the emergency department after ingesting 100 tablets of diphenhydramine 25 mg (46 mg/kg) with alcohol to self-harm. She experienced two generalized tonic-clonic seizures en route to the hospital. Initial vital signs were: pulse of 105 bpm, blood pressure of 141/101 mmHg, oxygen saturation of 100 % by mask ventilation, and temperature of 95.9 °F. She was intubated for airway protection. Laboratory results revealed hypokalemia (2.3 mmol/L), elevated lactate (10.4 mmol/L), and serum ethanol concentration of 187 mg/dL. A whole blood diphenhydramine concentration, taken six hours post-presentation, was elevated at 7324.9 ng/mL (therapeutic levels: 25–112 ng/mL). An electrocardiogram showed sinus tachycardia, heart rate 116 beats/minute, PR interval of 54 ms, QRS duration of 152 ms, and new LBBB. The patient received 150 mEq of sodium bicarbonate and a repeat electrocardiogram demonstrated sinus tachycardia and resolution of LBBB. During her hospital course, she maintained normal sinus rhythm without conduction abnormalities. She was extubated the following day and transferred to a psychiatric unit two days later. Reversible LBBB can be observed due to various etiologies including sodium channel blockade. Our case demonstrates that patients who develop a new LBBB after diphenhydramine overdose can be treated with standard therapy, including sodium bicarbonate.

## Highlights

• Diphenhydramine is a first-generation antihistamine with sodium channel blocking properties.
• A left bundle branch block can be observed after diphenhydramine overdose and can represent sodium channel blockade.
• New left bundle branch block after diphenhydramine overdose can be treated with standard care and sodium bicarbonate.

## 1 Introduction

Diphenhydramine is a first-generation antihistamine belonging to the ethanolamine class of histamine H_1_ receptor blocking agents [1]. It is commonly prescribed and sold over-the-counter and used often for its antihistamine and mild sedative effects. It is well absorbed after oral administration, readily crosses the blood brain barrier, and achieves peak plasma concentrations within 2–3 h [2]. In supratherapeutic or overdose settings, absorption can be prolonged due to the antimuscarinic effects on the gastrointestinal tract. It is lipid soluble and highly bound to plasma protein. It is primarily hepatically metabolized and renally excreted. Half-life is approximately 3–14 h [3] with duration of action up to 12 h [4]. Its estimated volume of distribution is 3–4 L/kg [3].

Diphenhydramine has several mechanisms of action including antagonism at histamine H_1_ receptors, antagonism at alpha-1 adrenergic receptors, antagonism at muscarinic receptors, and antagonism of sodium ion channels [2]. Toxicity commonly presents as signs and symptoms of an antimuscarinic toxidrome characterized by mydriasis, tachycardia, hyperthermia, dry mucous membranes, urinary retention, and altered mental status. Serious central nervous effects can include convulsions, ataxia, hallucinations, and coma [1]. Furthermore, diphenhydramine possesses membrane stabilizing properties similar to lidocaine [5] and has been used as a local anesthetic [6]. It prevents conduction of nerve impulses by binding with high affinity for the inactivated voltage-gated sodium channel thus increasing the threshold for electrical excitation and prolonging the action potential [7], [8].

Cardiotoxicity due to sodium channel blockade manifests as a wide-complex tachycardia and Brugada-pattern electrocardiographic changes which can be treated with sodium bicarbonate [9], [10]. Electrocardiographic findings typically show QRS prolongation with a rightward axis and “terminal R wave” [11], [12]. A left bundle-branch block (LBBB) morphology is rare in this setting but has been reported in overdoses by other sodium channel blocking agents [13]. We present a case of a reversible LBBB in a 35-year-old patient who presented after an intentional overdose of diphenhydramine.

## 2 Case report

**Fig. 1:**
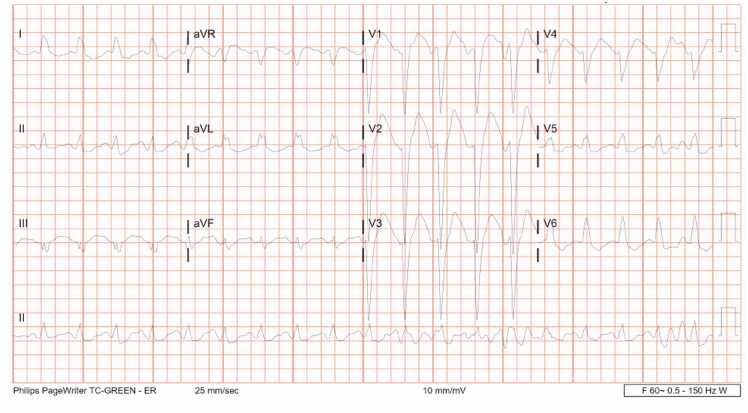

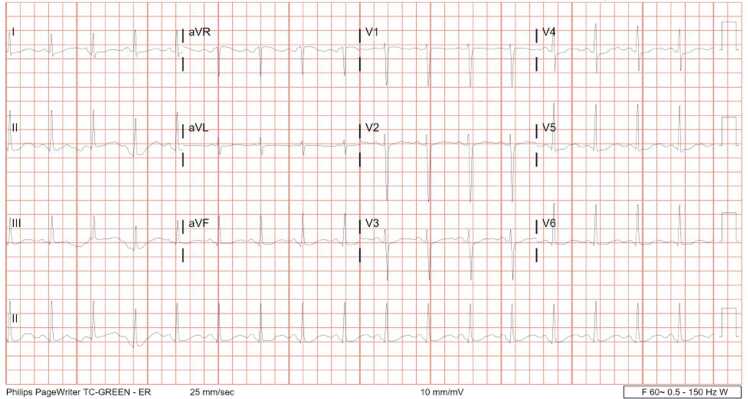
A. Initial 12-lead EKG with evidence of left bundle branch block. B. Repeat 12-lead EKG taken after 150 mEq sodium bicarbonate administration.

A 35-year-old woman with history of alcohol use disorder and depression with prior suicide attempts presented to the emergency department (ED) for altered mental status starting one hour prior to arrival. She reported to her mother that she ingested 100 tablets of diphenhydramine 25 mg (46 mg/kg) and alcohol in a self-harm attempt. En route, she had two generalized tonic-clonic convulsions and received 10 mg of midazolam intramuscularly by paramedics. On arrival, vital signs were blood pressure of 141/101 mmHg, pulse of 105 beats/minute, oxygen saturation of 100 % by mask ventilation, and temperature of 95.9 °F. She was noted to be obtunded and underwent rapid sequence intubation for airway protection using 20 mg etomidate and 100 mg rocuronium. Initial labs were notable for hypokalemia to 2.3 mmol/L, bicarbonate of 28 mmol/L, lactate of 10.4 mmol/L, and serum ethanol concentration 187 mg/dL. Confirmatory urine drug immunoassay was positive for benzodiazepines and cannabinoids. A whole blood diphenhydramine concentration, sent approximately six hours after her presentation, was 7324.9 ng/mL (therapeutic level 25–112 ng/mL). A 12-lead electrocardiogram (EKG) following intubation showed sinus tachycardia, rate 116 beats/minute, with PR 54 ms, QRS 152 ms, QT/QTc 301/319 ms, and a new LBBB pattern that was not present on her previous study from eight months prior (Fig. 1A). She subsequently received a total of three ampules of 8.4 % 50 mEq sodium bicarbonate to treat presumptive sodium channel blockade. Repeat 12-lead EKG following treatment showed sinus tachycardia, rate 101 beats/minute, with PR 132 ms, QRS 90 ms, QT/QTc 420/545 ms, and resolution of the LBBB (Fig. 1B). She was then admitted to the intensive care unit. Repeat 12-lead EKGs performed during admission demonstrated normal sinus rhythm with a prolonged QTc (500–530 ms) but no conduction abnormalities. Continuous telemetry monitoring was also unremarkable. A computed tomography (CT) pulmonary angiogram was within normal limits. She was extubated the day after presentation and transferred to an inpatient psychiatric unit two days later.

## 3 Discussion

This case report highlights a patient who developed a reversible LBBB after a massive diphenhydramine ingestion. The patient’s LBBB resolved after sodium bicarbonate treatment and aggressive supportive care.

Diphenhydramine toxicity often presents clinically with an antimuscarinic toxidrome with cardiotoxicity manifesting after massive ingestions, defined as greater than 20 mg/kg [14], [15]. It has local anesthetic and membrane stabilizing properties due to inhibition of voltage-gated sodium channels. Cardiac sodium channel blockade is more commonly associated with right sided conduction effects as the right-sided intraventricular conduction system has higher susceptibility to sodium channel blockade than the left bundle. In addition, the right bundle branch has a longer refractory period and slowing of the depolarization can result in the observed right bundle branch block pattern on EKG [16].

Several case reports demonstrate wide-complex tachycardia after diphenhydramine overdose, but a LBBB morphology is rarely described.

One published case report observed a LBBB after diphenhydramine overdose [14]. The patient was a 13-month-old who ingested 100–150 mg of diphenhydramine hydrochloride and subsequently developed convulsions with an EKG demonstrating complete LBBB. The patient received phenobarbital intramuscularly and underwent endotracheal intubation due to respiratory arrest. Five hours later, a repeat EKG showed sinus rhythm with nonspecific ST-T wave changes. The authors concluded that the cardiac dysrhythmia observed was due to hypoxia secondary to sedation, which resolved after mechanical ventilation to improve myocardial oxygenation. Interestingly, the LBBB resolved with supportive measures and no administration of sodium bicarbonate was documented. A diphenhydramine concentration was not obtained. There are two other case reports that demonstrate resolution of diphenhydramine-induced conduction abnormalities after administration of sodium bicarbonate [11], 17].

The occurrence of a LBBB may suggest different underlying mechanisms compared to typical sodium channel blockade effects on cardiac conduction. An article that presented a case report and literature review described a LBBB after a lamotrigine overdose and postulated that toxic LBBB might be linked to factors such as increased ventricular ischemia or irritability; these factors can also occur during severe illness, stress, and exercise [13]. A LBBB has also been described in the setting of carbon monoxide poisoning likely related to impairment of oxygen transport, which could provide insight on an alternative mechanism not related to sodium channel blockade alone [18]. Additionally, acidemia, often observed after convulsions, can promote conduction delays and dysrhythmias [19]. It is interesting that our patient’s conduction abnormality was responsive to sodium bicarbonate therapy, which would indicate sodium channel blockade effects. However, it is also possible that by supporting the patient’s respiratory status through mechanical ventilation and improving her acidemia, the LBBB would have naturally resolved with or without sodium bicarbonate administration.

This case has several limitations. There was no documented initial blood gas to assess for the degree of expected acidemia in the setting of recent convulsions when the patient first presented to the ED. Tissue hypoxia in the setting of convulsions and endotracheal intubation may not be evident by pulse oximetry alone and may have been contributory to the patient’s abnormal cardiac conduction as previously mentioned above. While the morphology and function of the patient's heart was normal on EKG, she did not undergo follow up electrophysiologic testing after discharge to rule out an undiagnosed cardiac conduction, genetic, or functional abnormalities.

## 4 Conclusion

We report an otherwise healthy patient who developed a reversible LBBB treated with sodium bicarbonate therapy in the setting of a massive diphenhydramine overdose. We conclude that when approaching a poisoned patient who develops a new LBBB, diphenhydramine overdose should be considered and can be treated with standard therapy despite a possible multifactorial etiology.

## CRediT authorship contribution statement

**Richard Clark:** Writing – review & editing, Supervision. **Justin Seltzer:** Writing – review & editing, Supervision. **Martin Krause:** Writing – review & editing, Data curation. **Mina Ghobrial:** Writing – review & editing, Data curation. **Henrik Galust:** Writing – review & editing, Data curation. **Jeremy Hardin:** Writing – review & editing, Data curation. **Riku Moriguchi:** Writing – review & editing. **Kara Yeung:** Writing – original draft, Investigation, Formal analysis, Data curation.

## Declaration of Competing Interest

The authors declare that they have no known competing financial interests or personal relationships that could have appeared to influence the work reported in this paper.

## Data Availability

No data was used for the research described in the article.
